# Rural Life

**Published:** 1893-02

**Authors:** 


### RURAL LIFE.

Rural life, country life and farm life are synonymous terms. Each one means life in the country, and life in the country means a great deal more than mere existence. It is varied and abundant, full and complete. The earth and air are full of life, and both earth and air with pleasant sounds are rife. In the country are all varieties of fowls and animals. Among the serviceable, the horse—man's inseparable companion. Who does not admire his fine form, beautifully molded limbs, arched neck and long, flowing mane? Too much cannot be said in his favor. The mule, so well adapted by his strength to farm work. The donkey, whose slowness makes him a favorite with a novice in horsemanship. The ox, whether harnessed to the plow or drawing a heavy load, bends his neck beneath the weighty yoke, while his parting hoofs press into the ground, his nostrils dilate, and his eyes seem almost starting from their sockets, but he patiently performs his work.

Among the useful, the cow—one of the choicest blessings to man. The sheep, true type of innocence, its bitterest complaints only expressed by pitiful bleatings. The goat, useful for different purposes, and the hog, which is indispensable. Fish—a fish pond an ornament to any farm.

Among the domestic fowls, ducks and geese, their flesh an excellent food and their feathers good for beds and pillows. Chickens around every door. Guineas, their speckled plumage very beautiful, and there is something almost sublime in their single note. The turkey, crowning adjunct to the festal board! The peacock, and who does not admire his gorgeous beauty? Proud bird! Is it vain from a sense of its beauty? Among the house animals, the cat and dog.

Life in the country is life among living landscapes. Fields of grain, over which shimmering waves follow each other in quick succession, are spread out to feast the eye. Dark green fields of rustling corn delight both eye and ear. Fields of rank clover, where humming bees gather sweets all the day. Rich green pastures, where grazing flocks and herds roam in happy freedom. We hear on every hand the hum of labor; the whizzing and whirring of machinery; the creaking wagon, crack of whip and hello of the teamster; the bleating and lowing of flocks and herds and the tinkle of bells.

The forests make up a wonderful part of the beauty of the country. How delightful to roam into their depths, the home of the birds, and listen to their sweet music! To hear the soft zephyr sighing among the trees and catch the low, wailing sound as it dies away in the pine tops. To listen to the chirp and chant and songs of insects. To gather flowers and ferns. To watch the nimble squirrels skip from branch to branch of the trees, undisturbed by our almost noisless tread over the leaf-carpeted ground.

How sweet to wander at eventide across the meadow and down by the river's side, and there to sit and listen to the rushing, roaring river, and, on our homeward way, to stroll along the murmuring little stream that skirts the meadow, and return when twilight is deepening.

O give me a home in the country,
  Where the air is fragrant and free;
Where the first pure breathings of morning come
  In a gush of melody,
Where day steals away with a young bride's blush
  To the soft green couch at night.
And the moon throws o'er with a holy hush
  Her curtain of gossamer light.

